# The Efficacy of Different Laser Applications on Dentin Sealing in Preventing Discoloration Induced by Mineral Trioxide Aggregate

**DOI:** 10.3390/ma17051015

**Published:** 2024-02-22

**Authors:** Yesim Sesen Uslu, Burçin Arıcan Alpay, Pinar Sesen, Taha Özyürek

**Affiliations:** 1 Department of Restorative Dentistry, School of Dental Medicine, Bahçeşehir University, Istanbul 34349, Turkey; 2 Department of Endodontics, School of Dental Medicine, Bahçeşehir University, Istanbul 34349, Turkey; burcin.aricanalpay@bau.edu.tr (B.A.A.); taha.ozyurek@bau.edu.tr (T.Ö.); 3 Department of Prosthodontics, Faculty of Dentistry, Istanbul Kent University, Istanbul 34433, Turkey; pinar.sesen@kent.edu.tr

**Keywords:** MTA, dentin-bonding agent, Nd:YAG laser, Er YAG laser, Er Cr: YSGG laser, tooth discoloration, dentin tubule occlusion

## Abstract

The aim of this in vitro study was to evaluate the effect of the pre-application of a dentin-bonding agent and the application of different lasers on the prevention of tooth discoloration caused by mineral trioxide aggregate (MTA) in the presence of blood. Sixty extracted human anterior teeth were selected, with root lengths standardized to 10 mm and root canals shaped using Gates-Glidden drills #3 to #5. The samples were divided into six groups (*n* = 10): Group 1 with no surface treatment and Groups 2 to 6 with Optibond universal adhesive and Neodymium yttrium aluminum garnet (Nd:YAG), Erbium yttrium aluminum garnet (Er:YAG), Erbium-chromium-yttrium-scandium-gallium-garnet (Er:Cr:YSGG), and diode laser applications, respectively. Root canals were filled with fresh human blood, and ProRoot MTA and a collagen barrier were then placed. Color changes were measured with a spectrophotometer at 0, 7, 30, 90, and 180 days post MTA placement. Color differences (∆E) were analyzed using a two-factor mixed-design ANOVA with the Sidak method (*p* = 0.05). All treatment groups exhibited discoloration above the acceptability threshold. Although all treatment approaches exhibited less color change compared to the control group (*p* < 0.05), there was no significant difference among them in terms of preventing color change (*p* > 0.05). It was determined that none of the methods could guarantee 100% prevention of discoloration caused by MTA–blood contact.

## 1. Introduction

Regenerative endodontics (RET) is an area that explores the potential of biologically based procedures for the regeneration of damaged pulp, including dentin and other root structures, as well as for the recreation of pulp–dentin tissue [1]. In this treatment procedure, the aim is to restore the vitality of the tooth by thickening the root canal walls and ensuring the continuation of root development [2]. A biocompatible material, often MTA, is typically placed over the blood clot [3]. Nevertheless, in approximately 40% of RET cases, discoloration is observed due to the interaction between the blood clot—which is crucial for natural scaffold formation in the apical region—and the applied MTA [4,5,6]. The reasons for this tooth discoloration include not only blood–MTA contact but also the destabilization of bismuth oxide in contact with sodium hypochlorite [7]. These unacceptable color changes after dental treatments cause the treatment to be evaluated as esthetically unsuccessful. This is a primary concern causing esthetic discomfort among patients [8]. Hence, regenerative endodontic treatments consider not only the biological and functional aspects but also the potential esthetic considerations of the procedure [9,10].

In order to prevent tooth discoloration, different treatment methods that ensure the pretreatment occlusion of the dentin tubules have been used [11,12]. One of the oldest methods is sealing the dentin tubules of the coronal cavity with a bonding agent [12,13]. It has been shown that applying two layers of adhesive resin before MTA application can prevent tooth discoloration. However, the occlusive property of the adhesive resin was not fully effective at preventing the penetration of MTA into dentin tubules, which has paved the way for the investigation of new methods [11,13].

In the literature, dental laser application is another approach that yields satisfactory results for dentinal tubule occlusions, in addition to desensitizing agents [14]. Dental lasers such as Er:YAG, carbon dioxide (CO_2_), Nd:YAG, and diode lasers can occlude dentin tubules through the recrystallization or melting of dentin and are effectively used in the management of dentin hypersensitivity (DH). The application of a Nd:YAG laser operates by blocking dentin tubules and providing nerve analgesia, whereas Er:YAG lasers function by dissolving and vaporizing the surface layer of the dentin, resulting in the melting of the tubules [15,16].

While the literature contains many studies exploring color changes associated with regenerative endodontic treatments [3,7,17,18,19,20], there are exceedingly few studies that assess strategies for preventing such color changes [11,12,21,22]. The interaction of different laser applications with dentin may result in a decrease in discoloration. However, different laser applications have not yet been evaluated and compared with each other. The purpose of this in vitro study is to compare the effect of dentine tubule occlusion achieved with different lasers on preventing discoloration induced by MTA. The null hypothesis is that the different tubule occlusion treatments tested would not prevent such discoloration and would not differ from each other. The null hypothesis of this study is that there will be no difference among the effectiveness of different tubule occlusion applications on the prevention of tooth discoloration.

## 2. Materials and Methods

This study’s protocol received approval from its local ethics committee under the reference number 2023-05/05. A comprehensive power analysis executed using G*Power 3.1 software (Faul et al. [23]) and drawing upon insights from a comparable study [3] established a minimum required sample size of 10 participants for each experimental group in this investigation. This analysis established an alpha probability of error at 0.05 and targeted a statistical power of 0.95, considering a correlation among repeated measurements of 0.5 and an effect size of 0.25.

### 2.1. Tooth Selection

**Figure 1:**
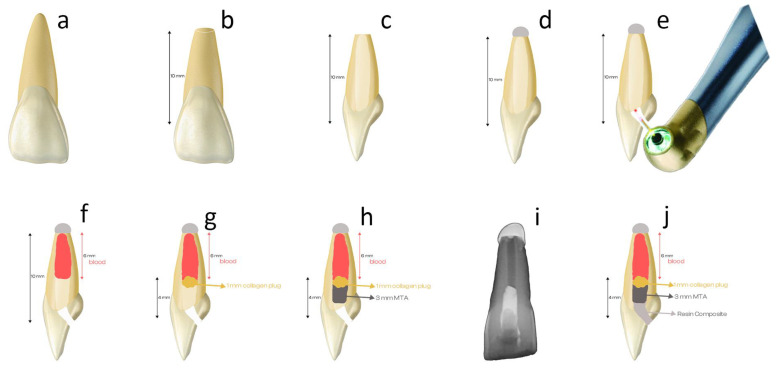
Illustration of the experimental stages and setup. (**a**) Schematic presentation of maxillary central incisor. (**b**) After cutting the tip of the tooth. (**c**) Shaping root canals with Gates-Glidden drills. (**d**) Covering the apex with composite restoration. (**e**) The laser tip was applied to the pulp chamber walls at a distance of 1 mm from the dentin surface. All laser applications were performed with a light guide moving back and forth over the surface of all dentin walls of the pulp chamber (the endodontic access cavity of the crown). (**f**) The blood inserted into the last 6 mm of the root canal. (**g**) Placing a collagen plug over the blood clot. (**h**) MTA placed over the plug. (**i**) Checking the position and thickness of the MTA using periapical X-rays. (**j**) The restoration of the coronal cavity with the resin composite.

Sixty maxillary permanent incisor teeth with mature apexes which were extracted due to periodontal reasons were examined using a dental microscope (OMS2380, Zumax, Suzhou, China) to identify any with cracks, fractures, or caries. Initial periapical radiographs were taken from the bucco-lingual and mesio-distal aspects to consider the root canal anatomy. Teeth with a single root and a single canal configuration (Vertucci Class I) for which the initial root angle was less than 10° were included in the study (Figure 1a). To achieve similarity in dentin tubule structures, teeth from female and male patients within the age range of 40–60 were utilized. Teeth with caries, old restorations, root canal treatment, unusual anatomical features, external or internal root resorption, post-core restoration, cracks, calcification, or initial discoloration were excluded from the study. The teeth were then kept in distilled water until the experiment.

### 2.2. Preparation of Specimens

The root tips were cut to ensure a standardized working length of 10 mm measured from the cementoenamel junction (CEJ) (Figure 1b). A traditional endodontic access cavity was prepared with a round bur. Gates-Glidden drills ranging from sizes #3 to #5 were utilized to instrument the entire length of the root (Figure 1c). An irrigation protocol was performed using 5 mL of 2.5% sodium hypochlorite and 5 mL of 17% EDTA, followed by rinsing with 5 mL of distilled water. Subsequently, the root canals were dried using sterile paper points, and a sterile cotton pellet was placed from the access opening down to the CEJ. The root ends were sealed with a Z350 resin composite (3M ESPE, St. Paul, MN, USA) (Figure 1d), and the access cavity was temporarily closed using Cavit (3M ESPE, St. Paul, MN, USA). All steps were carried out using a dental microscope (OMS2380, Zumax, Suzhou, China), and samples were kept in saline until the experiment began.

The teeth were taken from the saline solution, the cotton was extracted, and the same final irrigation protocol was applied once more. The root canals were then dried. Since the aim of this study was to observe and compare the preventive effect of different laser groups on tooth discoloration, the samples were divided into six groups: one control, one bonding, and four laser groups. Bonding application, which is the most common pre-application method in the literature, was chosen to compare the effectiveness of the laser groups. A control group was added to compare the effects of all groups. The details of the groups created accordingly are as follows: 

**Group I—control group:** the dentin walls of the pulp chamber were not sealed with a DBA (Optibond Universal (Kerr Corporation, Orange, CA, USA) and/or a laser.

**Group II—bonding group:** Optibond Universal bonding agent was utilized on the dentin walls of the pulp chamber with a self-etch approach. This process involved rubbing for 20 s, followed by 5 s of air drying and culminating with a 10 s polymerization.

**Group III—Nd YAG laser:** The dentin walls of the pulp chamber were irradiated with a Nd:YAG laser (Fotona, Ljubljana, Slovenia), using the following settings: 1 W of power, a 10 Hz frequency, and a pulse duration of 50 μs. This procedure was applied using a non-cooled handpiece equipped with a 300 μm optical fiber for a total duration of 60 s.

**Group IV—Er YAG laser:** The dentin walls of the pulp chamber were treated with a 2940 nm Er:YAG laser (Fotona, Ljubljana, Slovenia), using the following parameters: 50 mJ, 0.50 W, and a 10 Hz frequency. A cylindrical sapphire optical fiber tip, measuring 1.3 mm in diameter and 8 mm in length, was utilized with a non-contact H02 handpiece.

**Group V—Er,Cr:YSGG laser** (Waterlase Biolase, Biolase, Foothill Ranch, CA, USA): The laser tip was held onto the irradiated pulp chamber surface 1 mm from the dentine surface. The application was performed in hard-tissue mode using the MZ6 sapphire tip (600 µm diameter and 6 mm length), employing a non-contact approach. The settings were an energy level of 0.25 W, a 20 Hz frequency, and a pulse duration of 140 ms, with 10% air and 0% water.

**Group VI—970 nm diode laser** (SIROlaser Blue, Sirona Dental Systems, Bensheim, Germany): the laser tip was applied to the pulp chamber walls at a distance of 1 mm from the dentin surface with parameters of 970 nm, a power of 0.8 W, and a duration of 60 s.

All laser applications were performed with the light guide moving back and forth over the surfaces of the dentin walls of the pulp chamber (the endodontic access cavity of the crown). For all groups treated with a laser, the samples were treated in a non-contact manner by the same dentist (Y.S.U.) using the fiber optic tip. The application involved movements in the occlusoapical and mesiodistal directions. The application involved horizontal and vertical movements. (Figure 1e)

After applying dentinal tubule occlusion strategies, 0.2 cc of fresh human blood was taken from a systemically healthy researcher (T.Ö.) and placed into the root canal via an insulin syringe, ensuring it reached almost 4 mm below the cementoenamel junction (CEJ) (Figure 1f). To ensure the formation of a blood clot, 15 min was allowed to elapse, followed by the application of Spongostan (Cutanplast, Milan, Italy) over it (Figure 1g). Subsequently, ProRoot MTA (Dentsply Maillefer, Ballaigues, Switzerland) was placed in the coronal third of the root canal using a micro-apical placement system (MAP, Produits Detaires SA, Vevey, Switzerland) (Figure 1h). The thickness of the MTA was 3 mm, and it was placed 1 mm below the CEJ. The coronal cavity of each sample was then filled with a temporary filling material. The position and thickness of the MTA were verified using a periapical X-ray (Figure 1i). The specimens were stored for 24 h in an environment with 100% humidity at 37 °C to allow for the setting of the cement. The coronal cavity of each sample was then restored with a Z350 (3M ESPE, St. Paul, MN, USA) resin composite (Figure 1j). The specimens were maintained at a constant temperature of 37 °C and 100% humidity throughout the experiment.

### 2.3. Tooth Color Measurements

To create standardized experimental groups and ensure an equal and balanced distribution of teeth, the initial color values of all samples were measured before the experiment using a spectrophotometer (VITA Easyshade Advance 4.0, VITA Zahnfabrik, Bad Säckingen, Germany). In each color measurement, the L*a*b* coordinates were recorded, and the average values were calculated. With the recording of initial L*a*b* data, tooth color reference values were also determined based on the VITA Classical shade guide and the VITA 3D Master scale guide. To ensure uniformity and prevent discrepancies both within and among the groups, the teeth were distributed evenly across the experimental groups: 4 teeth with an A2 shade, 2 teeth with a B2 shade, and 4 teeth with an A3 shade [7,17].

The teeth were placed in front of a white background (a calibration tile adhering to Commission Internationale de l’Éclairage [CIE] standards with L*, 93.84; a*, 1.48; and b*, 3.76 values) for a color evaluation. The color measurements were focused on the central region of the buccal surface of each tooth. The spectrophotometer’s tip was carefully positioned to fully engage with the buccal surface’s flattest area, ensuring that measurements were confined to the central region. A single researcher (Y.S.U.) was responsible for all evaluations, checking the device’s tip placement before each assessment. The spectrophotometer was calibrated as per manufacturer guidelines before measuring each sample group, with three repeated measurements conducted for each sample by the same researcher.

Color measurements were performed using the CIE L*a*b* color space system. In this system, the L* value represents the object’s lightness, varying from white (0) to black (100). The a* value indicates the color’s position between green (−) and red (+), typically extending from −70 (green) to +70 (red). The b* value quantifies the color on a scale from blue (−) to yellow (+), with values ranging from −80 (blue) to +100 (yellow).

After the initial color assessment (T0), the teeth were stored in artificial saliva in an incubator at 37 °C, imitating the oral environment and aging process. The saliva was replaced every week. Further color evaluations were carried out at intervals of (T1) 7, (T2) 30, (T3) 90, and (T4) 180 days for a comparison with the initial L*a*b* values (baseline). All measurements were conducted using the CIEDE2000 (ΔE00), and the ΔE00 was determined using the following formula [24,25]:

$${{∆E}_{00}=\left[{{\left(\frac{∆L^{\prime }}{K_{L}S_{L}}\right)}^{2}+\left(\frac{∆C^{\prime }}{K_{C}S_{C}}\right)}^{2}+{\left(\frac{∆H^{\prime }}{K_{H}S_{H}}\right)}^{2}+R_{T}\left(\frac{∆C^{\prime }}{K_{C}S_{C}}\right)\left(\frac{∆H^{\prime }}{K_{H}S_{H}}\right)\right]}^{1/2}$$

### 2.4. Statistical Analysis

A statistical analysis was conducted using SPSS software, version 21. For each variable, descriptive statistics were calculated and presented as the ‘mean ± standard error of mean (SEM).’ Prior to hypothesis testing, data were evaluated for normality and homogeneity of variance using the Shapiro–Wilk and Levene tests, respectively. A two-factor mixed-design ANOVA, as part of the General Linear Model for repeated measures, was utilized to analyze the data. This model included ‘Group’ and ‘Time’ as the main factors and their interaction term (Group*Time). Where Mauchley’s test showed a sphericity assumption breach, the Greenhouse–Geisser correction was applied. Significant interaction effects were further explored using a simple effect analysis with a Sidak adjustment for post hoc tests. Statistical significance was established at an alpha value of 0.05.

## 3. Results

**Figure 2:**
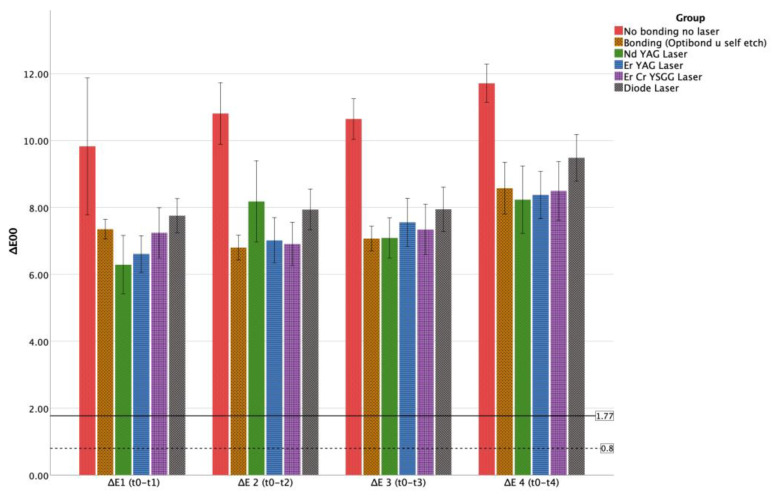
Comparison of the mean ΔE values of the groups for each treatment step with a graph (dotted lines represent AT = 1.77 and PT = 0.8) [25].

Mean and standard error values of the color change (ΔE) are presented in Table 1 and Figure 2. As shown in Table 1, discoloration was observed in all groups in all time intervals. While the highest degree of discoloration was observed in the control (*no laserno bonding*) group (*p* < 0.05), no significant difference was observed between the experimental groups regarding ΔE values (*p* > 0.05).

The ΔE values for experimental groups from T0 to T180 ranged from 6.29 to 11.71 (Table 1). The highest ΔE values for each group were obtained at T180, which is statistically significant from the other time intervals (*p* < 0.05). The lowest ΔE values for the initial (T0) and final (T180) experiment days were obtained in the Nd YAG laser group. 

**Figure 3:**
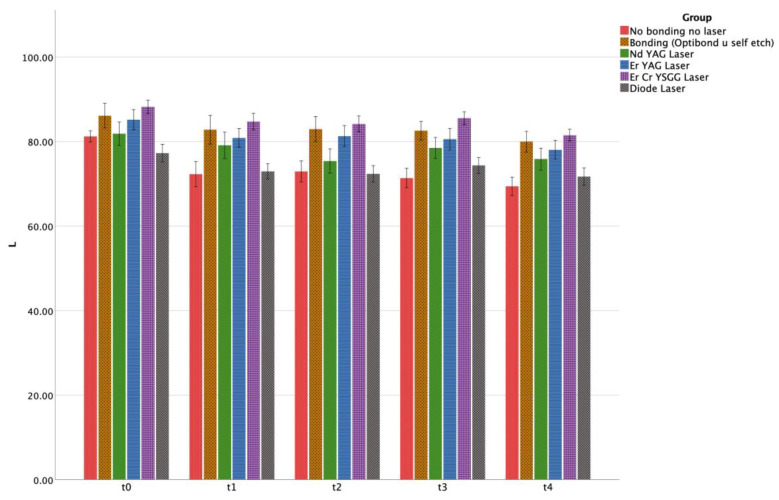
Graphical representation of the mean value of L (lightness) across all groups for each time interval.

**Figure 4:**
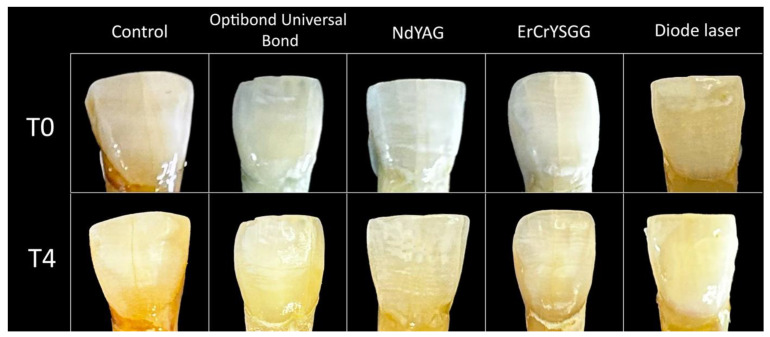
Sample examples from each group, illustrating the discoloration from time intervals T0 to T4.

For each time period, L*a*b* values were also calculated. Mean and standard error values of luminosity (ΔL*) are shown in Table 2 and Figure 3. A notable decline in enamel luminosity was observed (Figure 4).

## 4. Discussion

Tooth discoloration presents a major esthetic challenge often faced in the aftermath of RET. Despite the investigation of various treatment approaches in the literature to prevent tooth discoloration associated with RET, a definitive and universally successful treatment option to completely prevent discoloration has not yet been established. This is the first study which evaluates the role of different laser applications in preventing discoloration induced by MTA. The results demonstrate that the application of a universal adhesive as well as NdYAG, ErYAG, ErCr:YSGG, and 970 nm diode laser treatments to the dentin of the pulp chamber reduced coronal discoloration for all time intervals compared to a control group, although no statistical differences were found (*p* < 0.001). Consequently, the null hypothesis stating that there is no significant statistical difference among the experimental groups is accepted.

After biomechanical preparation with endodontic tools, root canals are also exposed to the chemical effects of irrigation solutions such as NaOCl [17]. It penetrates the dentin matrix (approximately 300 µm) and can disrupt collagen fibers. Even after drying, some residue of sodium hypochlorite may remain on the dentin walls [26,27]. When MTA is applied, it comes into contact with the remaining sodium hypochlorite and the amino acids of the disrupted collagen [17]. Bismuth oxide, a radiopacifier in MTA, has been reported to be a factor contributing to tooth color changes. The process of oxidation in bismuth oxide is believed to cause an instability in the material’s oxygen content. This instability may lead to a reaction with carbon dioxide, forming bismuth carbonate, which is implicated in the discoloration of teeth [28,29]. It is also suggested that the interaction between bismuth oxide and dentin collagen is another contributing factor. Moreover, the discoloration issue may be exacerbated by the MTA slurry’s interaction with blood during hydration [18,30].

The CIE 2000 formula is the most current method used for calculating color differences, and due to its superior performance and better compatibility with visual evaluations compared to CIELAB, it is recommended by the CIE [31,32]. Regardless of the outcomes of descriptive and analytical statistics, it has been reported that for the interpretation of their relevance to real-life situations, clinical and research findings should be compared with perceptibility and acceptability thresholds [32]. The PT (perceptibility threshold) refers to a scenario in which observers can perceive a color difference between two objects, whereas the AT (acceptability threshold) denotes a situation in which the observed color difference, though noticeable, is still considered acceptable. In the context of the CIEDE2000 system, the threshold values have been identified as 0.8 for the perceptibility threshold (PT) and 1.8 ΔE00 for the acceptability threshold (AT). Furthermore, a new classification for the degree of color mismatch has been proposed: moderately unacceptable (1.8 < ΔE00 ≤ 3.6), clearly unacceptable (3.6 < ΔE00 ≤ 5.4), and extremely unacceptable (>5.4). Based on these insights, it was observed in the current study that the values obtained for all tested groups, across all time intervals, were in the extremely unacceptable range [32].

For each of the experimental groups, there was a notable decline in enamel luminosity at the six-month mark when compared to the baseline measurements. This decrease in enamel luster across all groups is indicative of a gradual darkening of the specimens, implying significant alterations in tooth discoloration.

In this study, to ensure standardization, initial color measurements were conducted, and with the recording of the first L*a*b* values, tooth color reference values were determined according to the VITA 3D Master guide. To prevent intra-group and inter-group discrepancies, teeth were evenly allocated, ensuring the formation of experimental groups with a balanced distribution. When evaluating the literature, it is observed that studies on tooth discoloration due to RET have considered various duration times. In our current study, we conducted our color measurements in a manner similar to Jesus et al. [17]: an initial color measurement post restoration (T0), followed by measurements at 1 week (T1), 1 month (T2), 3 months (T3), and 6 months (T4). Unlike this current study, de Jesus et al. also evaluated color changes at 365 days [17]. When each experimental group was evaluated individually, it was observed that a statistically significant discoloration became apparent after 6 months. Similar to our findings, de Jesus et al. [17] also found, in their study, the greatest color differences at 180 days and 365 days. Within the same experimental group, the color change over time did not follow an increasing pattern throughout the experiment [33]. This could be attributed to the multi-colored nature of the tooth structure. Additionally, it should be noted that over certain periods of time, cements release a higher amount of chemical compounds. This factor could potentially affect the esthetic behavior of MTA-based cements [34]. Based on this, it can be concluded that it is necessary to follow the long-term consequences of coloration that may occur after MTA use.

In the literature, the effect of pre-applying a dentin-bonding agent to the dentin walls of the pulp chamber was evaluated for the purpose of preventing color changes caused by MTA [12,13,22]. In the current study, a universal adhesive was applied using a self-etch approach and found to be effective at preventing color changes for all time intervals compared to the control group. While this finding is consistent with the literature, there was no statistical difference observed when compared with other preventive strategies. However, it is important to mention that although the application of a universal bonding agent reduces the color change caused by RET, color alterations exceeding the clinically acceptable threshold were observed after six months, indicating that it did not completely prevent discoloration. In the current study, the dentin-bonding agent was applied not to the entrance of the root canal but to the dentin walls of the tooth’s coronal part. Applying the universal adhesive (as a color-preventive treatment) to the dentin surrounding the root canal may jeopardize the success of RET [12].

In the treatment of dentin hypersensitivity, various lasers are currently employed, including Nd:YAG, Er:YAG, Er Cr:YSGG, and diode lasers [35]. The effectiveness of Nd:YAG lasers in providing efficient tubule occlusion and penetration depth for the treatment of DH was reported [36]. Additionally, it has been noted that the application of a Nd:YAG laser with appropriate parameters can prevent unwanted side effects (cracks and pulp damage). Lan et al. [37] reported that exceeding a critical power value of 1.5 W with a Nd:YAG laser can lead to irreversible damage on the tooth surface. White et al. [38] recommended the use of a Nd:YAG laser at 1 W of power or less to protect the pulp. In the current study, due to these reasons, the Nd:YAG laser was used at a power of 1 W.

The Er:YAG laser, the highest water absorption peak at a wavelength of 2940 nm, which is effectively absorbed by dental hard tissues, is a highly favored laser type for the treatment of these tissues [39]. It is believed that the effectiveness of the Er:YAG laser in occluding dentin tubules is due to the absorption of laser irradiation by water molecules within hydroxyapatite, leading to dentin ablation and subsequently causing the melting and recrystallization of the dentin [39,40]. Zhuang et al. [35] investigated the effect of Er:YAG laser application on dentin tubule occlusion and intrapulpal temperature increase and its impact on pulp using different parameters (0.5 W/50 mJ/10 Hz, 1 W/50 mJ/20 Hz, 2 W/100 mJ/20 Hz, and 4 W/200 mJ/20 Hz). As a result, they found the 0.5 W and 50 mJ setting to be effective for dentin tubule occlusion. Therefore, in this study, the Er:YAG laser was used with parameters of 0.5 W, 50 mJ, and 10 Hz [35]. 

The other member of the erbium family, the 2780 nm Er,Cr:YSGG laser, due to its high absorption in water, causes the evaporation of dentinal tubular fluid and the precipitation of insoluble salts within the dentin tubules, thereby leading to the occlusion of dentin tubules [41]. Gholami et al. [42] used a laser at 0.25 W in their study and reported the formation of occlusion in dentin tubules as a result of melting occurring in the peritubular dentin tubules [42]. Aranha and Eduardo [43] compared the effects of an Er,Cr:YSGG laser at various parameters and reported that a power setting above 0.75 W resulted in a certain degree of carbonization. In our study, the parameters for the Er,Cr:YSGG laser were therefore selected as 0.25 W at 20 Hz.

Generally, diode lasers produce a range of wavelengths from 720 to 904 nm which are close to the absorption peak of specific chromophores like hemoglobin yet absorbed less by the hard tissues of the tooth [44,45]. When the dentin surface is exposed to a laser, the energy from the laser is transformed into heat. This heat then leads to the sealing of the dentin tubules through the recrystallization of the dentin’s mineral components (the photothermal effect) [46]. Meng et al. [44] utilized a 980 nm diode laser at 0.8 W and 1 W in their study and found it to be highly effective at occluding dentin tubules. However, based on increases in intrapulpal temperature and the results from an animal study model, they indicated that using the laser at 0.8 W is a safer option for treating dentin hypersensitivity [44]. For these reasons, and to better reflect the clinical situation, we applied the diode laser at a power of 0.8 W in our study.

The main significant limitation of this study is that it evaluated the secondary effect of lasers on discoloration rather than their direct occluding effect on dentin tubules. Therefore, it is not feasible to directly compare the current study with others that specifically evaluated the effect of covering dentin tubules. In the literature, we found only two studies that assess the impact of laser-induced dentin tubule occlusion on preventing discoloration. Fundaoğlu et al. [11] evaluated the preventive efficacy against discoloration caused by a triple antibiotic paste of three dentin tubule occlusion methods (a dentin-bonding agent, a desensitizing agent, and a Nd:YAG laser). They reported no significant difference in preventing discoloration among the treatments after 21 days [11]. Similarly, in the current study, all treatments were equally effective at preventing discoloration, and clinically perceptible color changes were observed in all experimental groups [11]. Ateş and Aydın [21] similarly added an Er:YAG laser experimental group in their study on preventing discoloration caused induced RET. They compared the effectiveness of a desensitizing agent, a bonding agent, a Nd:YAG laser, and an Er:YAG laser in preventing discoloration. In their study, which utilized calcium silicate-based Biodentin, discoloration was evaluated before treatment, after treatment, and at 7, 30, and 90 days post treatment. They found no difference in preventing color changes among the applied treatments in the outcome of their study [21].

The current findings suggest that the treatment methods used may have a potential effect on preventing discoloration, yet the lack of a difference between them indicates the need for further research with varied setting parameters. Additionally, the biological properties of laser irradiation, which can only be fully assessed under in vivo conditions, highlight the necessity of clinical follow-ups. There is a need for more comprehensive in vitro and in vivo studies.

## 5. Conclusions

Within the limitations of this in vitro study, different laser systems had promising results in preventing discoloration. However, no significant difference was detected in the efficacy of preventing tooth discoloration between the irradiation of the pulp chamber with lasers of different wavelengths prior to regenerative endodontic treatment and the application of a universal bonding agent. Additionally, the highest color change values were obtained at 180 days, which shows the importance of long-term analyses to obtain more reliable results in discoloration studies. It is important to note that none of the discoloration prevention treatments were found to be 100% effective. Considering ease of access and cost factors, the application of a universal adhesive may be preferable. However, it should be kept in mind that with laser systems, different settings and parameters laser systems may result in better consequences. Future in vivo studies considering the use of lasers with different parameters are needed.

## Figures and Tables

**Table 1:** Mean and standard error values of the color change values (ΔE00) for each time interval. ∆E values indicate color changes from the baseline measurement to various time intervals.

Group	n	ΔE1 (T0–T1) Mean ± SEM	ΔE2 (T0–T2) Mean ± SEM	ΔE3 (T0–T3) Mean ± SEM	ΔE4 (T0–T4) Mean ± SEM	Group	*p* Time	Group*Time
**Control**	10	9.83 ± 2.05 ^a,B^	10.81 ± 0.92 ^a,B^	10.64 ± 0.61 ^a,B^	11.71 ± 0.57 ^a,A^	<0.001	0.002	0.977
**DBA**	10	7.35 ± 0.29 ^b,B^	6.8 ± 0.37 ^b,B^	7.07 ± 0.37 ^b,B^	8.58 ± 0.77 ^b,A^			
**Nd YAG Laser**	10	6.29 ± 0.87 ^b,B^	8.18 ± 1.22 ^b,B^	7.09 ± 0.6 ^b,B^	8.23 ± 1.01 ^b,A^			
**Er YAG Laser**	10	6.61 ± 0.55 ^b,B^	7.02 ± 0.68 ^b,B^	7.55 ± 0.72 ^b,B^	8.37 ± 0.7 ^b,A^			
**Er Cr YSGG Laser**	10	7.24 ± 0.75 ^b,B^	6.91 ± 0.65 ^b,B^	7.34 ± 0.75 ^b,B^	8.49 ± 0.88 ^b,A^			
**Diode Laser**	10	7.75 ± 0.51 ^b,B^	7.94 ± 0.61 ^b,B^	7.95 ± 0.66 ^b,B^	9.48 ± 0.7 ^b,A^			

Different letters in the same column and row show statistical significance (*p* < 0.05).

**Table 2:** Mean and standard error values of lightness (ΔL), a, and b for each time interval.

Measurement	Time	Mean ± SEM	Median (Min.–Max.)
L	T0	83.3 ± 1	84.4 (62.35–99.6)
	T1	78.78 ± 1.2	78.93 (60.65–95.95)
	T2	78.18 ± 1.15	79.43 (61–97.4)
	T3	78.8 ± 1.06	81.1 (61.6–91.75)
	T4	76.08 ± 1.02	77.2 (63.05–90.05)
a	T0	5.78 ± 0.38	5.25 (0.3–14)
	T1	2.6 ± 0.32	1.88 (0.05–10)
	T2	1.88 ± 0.16	1.63 (0.25–5.95)
	T3	1.6 ± 0.15	1.28 (0–5.1)
	T4	1.33 ± 0.12	1.08 (0.15–3.9)
b	T0	42.68 ± 0.68	42.58 (30.9–51.1)
	T1	28.84 ± 0.77	27.98 (16.2–41.15)
	T2	28.59 ± 0.7	27.9 (15.1–40.1)
	T3	28.58 ± 0.7	28.05 (17.2–42.3)
	T4	27.18 ± 0.66	26.88 (16.9–41)

## Data Availability

The study materials and data can be requested in writing from the corresponding author.
